# Characterization of NOD-like receptor-based molecular heterogeneity in glioma and its association with immune micro-environment and metabolism reprogramming

**DOI:** 10.3389/fimmu.2024.1498583

**Published:** 2025-01-15

**Authors:** Chunlin Lu, Huihao Ma, Jie Wang, Fei Sun, Mingyang Fei, Ying Li, Jing Liu, Bin Dong

**Affiliations:** ^1^ Department of Neurosurgery, First Affiliated Hospital of Dalian Medical University, Dalian, China; ^2^ Department of Stem Cell and Clinical Research, First Affiliated Hospital of Dalian Medical University, Dalian, China; ^3^ Department of Neurosurgery, Xinhua Hospital Affiliated to Dalian University, Dalian, China; ^4^ The Administration center, First Affiliated Hospital of Dalian Medical University, Dalian, China

**Keywords:** NLR pathway, GBM, immune microenvironment, metabolism reprogramming, clinical heterogeneity, TRIP6

## Abstract

**Background and purpose:**

The characteristics and role of NOD-like receptor (NLR) signaling pathway in high-grade gliomas were still unclear. This study aimed to reveal the association of NLR with clinical heterogeneity of glioblastoma (GBM) patients, and to explore the role of NLR pathway hub genes in the occurrence and development of GBM.

**Methods:**

Transcriptomic data from 496 GBM patients with complete prognostic information were obtained from the TCGA, GEO, and CGGA databases. Using the NMF clustering algorithm and the expression profiles of NLR genes, these 496 GBM patients were classified into different clinical subtypes. The pathway activity of NLR and the immune micro-environment characteristics were then compared between these subtypes. A novel and accurate NLR expression profile-based prognostic marker for GBM was developed using LASSO and COX regression analysis.

**Results:**

Based on the NLR gene expression profile, GBM patients were accurately divided into two clinical subtypes (C1 and C2) with different clinical outcomes. The two groups of patients showed different immune microenvironment characteristics and metabolic characteristics, which might be the potential reason for the difference in prognosis. Differential expression and enrichment analyzes revealed intrinsic gene signature differences between C1 and C2 subtypes. Based on the differential expression profiles of C1 and C2, prognostic molecular markers related to NLR were developed. The AUC value of the 3-year ROC curve ranged from 0.601 to 0.846, suggesting its potential clinical significance. Single-cell sequencing analysis showed that the NLR gene was mainly active in myeloid cells within GBM. The random forest algorithm identified the crucial role of TRIP6 gene in NLR pathway. Molecular biology experiments confirmed that TRIP6 was abnormally overexpressed in GBM. Knockdown of TRIP6 gene can significantly inhibit the proliferation and migration ability of GBM cells.

**Conclusion:**

The NLR signaling pathway plays a critical role in regulating immune microenvironment and metabolism reprogramming of GBM. TRIP6 is a potential hub gene within the NLR pathway and affects the malignant biological behavior of GBM cells.

## Introduction

The clinical outcomes of patients with glioblastoma (GBM) are frequently very poor. The unique pathological features of GBM patients make it difficult for them to benefit from surgery or chemotherapy, and the 5-year survival rate is extremely low (1). The incidence of glioblastoma is significantly higher in men than in women. Most primary glioblastomas are more likely to occur in men, while secondary glioblastomas are more common in women (2). The combination of genetic and environmental factors leads to the abnormal expression of oncogenes and tumor suppressor genes, which may be the potential cause of glioblastoma. Despite the rapid progress of the multidisciplinary collaborative treatment model, the prognosis of glioblastoma patients has not been significantly improved, and the median survival is still difficult to exceed 3 years (3). In addition, most patients have poor survival outcomes after surgery, which brings a heavy economic burden to society. There is an urgent need to develop prognostic biomarkers for GBM and explore the potential mechanism of GBM pathogenesis and potential therapeutic targets.

NOD-like receptor (NLR) signaling pathway is a well-known star signal in the human innate immune response system (4, 5). Furthermore, NLRs are well-known members of the pattern recognition receptor family and play a crucial role in recognizing pathogen-associated molecular patterns and damage-associated molecular patterns (6). Abnormal NLR signaling can impact various downstream pathways, such as NF-κB, stress kinases, interferon response factors (IRFs), inflammatory caspases, and autophagy. This dysregulation can contribute to tumor development and progression (4). Abnormal expression of NLRP3 in GBM may promote biological processes such as invasion, migration, proliferation, anti-apoptosis, and epithelial-mesenchymal transition by activating the AKT pathway. The use of NLRP3 inhibitors such as β-hydroxybutyrate may have therapeutic potential in treating GBM patients (7).

In summary, NLR signals are closely related to the occurrence, development and prognosis of GBM. In this study, the NLR expression profile of GBM patients was analyzed by bioinformatics methods to reveal the important role of NLR signal in predicting clinical heterogeneity, clinical prognosis and tumor immune microenvironment. Single-cell sequencing further clarified the characteristics of NLR signaling in different cell types of GBM. In a variety of NLR signals, we found a new hub gene TRIP6, which might regulate a variety of malignant biological behaviors of GBM.

## Methods

### Sample source and NLR gene source

The transcriptomic sequencing data for GBM patients in this study were sourced from the following cohorts: TCGA-GBM, CGGA-GBM, GSE7696 (8), and GSE83300 (9). After excluding patients without follow-up information, a total of 496 GBM samples with complete prognostic features were retained. Given that data from different platforms might have batch effects, we applied the classical SVA method to correct for these batch effects to meet the requirements for subsequent bioinformatics analyses (10, 11). The single-cell sequencing data for this study was sourced from the GSE138794 cohort (12). The NLR genes were obtained from the KEGG database (KEGG NOD-LIKE RECEPTOR SIGNALING PATHWAY), which includes a total of 62 classic genes (**Table 1**).

**Table 1:** 62 NLR gene list.

TRIP6	CCL2	CARD8	MAPK13	SUGT1	CARD9	HSP90AA1	NAIP
CASP8	XIAP	CCL7	CASP5	MAPK3	MAPK8	PYCARD	BIRC2
CHUK	CCL5	MEFV	IKBKB	PSTPIP1	MAPK14	TAB2	CXCL2
TAB3	NOD1	CXCL8	NOD2	NFKB1	MAPK12	NLRC4	HSP90AB1
CXCL1	CARD6	MAPK9	MAP3K7	NFKBIB	IL18	IL1B	TAB1
RIPK2	CCL13	MAPK10	CASP1	NFKBIA	RELA	HSP90B1	IL6
TNF	CCL11	MAPK11	CARD18	MAPK1	PYDC1	NLRP3	
BIRC3	CCL8	ERBIN	TNFAIP3	IKBKG	TRAF6	NLRP1	

### Single-cell data analysis

The Read10X function was used to read single-cell data, DoubletFinder was employed to remove doublets. A series of bioinformatics algorithms, such as NormalizeData, FindVariableFeatures, and ScaleData, were used for normalization and analysis (13–15). Used the RunUMAP or RunTSNE functions for dimensionality reduction. Annotate the cells based on the following markers (12, 16, 17): “Astrocyte” = c(“ID3”, “AQP4”, “CST3”, “ID4”, “PON2”), “Endothelial_cell” = c(“CLDN5”, “IGFBP7”, “CTGF”, “IFI27”, “ITM2A”), “Myeloid” = c(“CCL3”, “CCL4”, “CD74”, “SPP1”, “C1QB”), “Neoplastic_cell” = c(“SEC61G”, “PTPRZ1”, “NOVA1”, “SOX4”, “PTN”), “Oligodendrocyte” = c(“PLP1”, “PTGDS”, “APLP1”, “CNP”, “ERMN”). Evaluate the NLR scores for each cell using six algorithms: Add, AUCell, UCell, singscore, ssgsea, and scoring (18). “Scoring” was the total score obtained by combining the scores from the previous five algorithms (19).

### Bulk RNA sequencing data analysis

The NMF algorithm was used to classify GBM patients into different clinical subtypes. The GSVA algorithm was employed to evaluate the NLR scores for each GBM patient. Kaplan-Meier analysis was utilized to reveal prognostic differences between the subtypes. Various immune microenvironment prediction algorithms, including ESTIMATE, TIMER, Cibersort, QUANTISEQ, MCPCOUNTER, XCELL, and EPIC, were used to uncover differences in immune cell infiltration between the subtypes (20). From previous literature (21), we collected classic immune checkpoint-related genes and analyzed the differences in immune checkpoint expression between different subtypes, as well as the association of these immune checkpoints with GBM prognosis. The oncopredict function was used to predict the potential drug sensitivity for each GBM patient and analyze the potential beneficial drugs for different subtypes. This analysis was facilitated using the Sangerbox platform (22). Differentially expressed genes between different subtypes were analyzed, and functional enrichment analysis was performed on these genes.

LASSO-Cox regression analysis (23) was applied to further establish a prognostic model based on above differentially expressed genes (24). Firstly, we integrated the GBM data from the TCGA and GEO databases, and then randomly selected 50% of the samples as the training cohort for the prognostic model. The remaining 50% of the samples were used as the internal validation cohort 1 of the prognostic model. In addition, we used all GBM data from the TCGA and GEO databases as the internal validation cohort 2. The GBM data from the CGGA database were used by us as an external validation cohort during the model development process. In short, we used a combination of training sets, internal validation sets, and external validation sets to make our prognostic model construction more stable. Kaplan-Meier survival curves and ROC plots were performed for each cohort to highlight the stability of the prognostic marker (25).

Additionally, we also aimed to identify hub genes within the NLR signaling pathway, and investigate their crucial roles. Therefore, we used the random forest machine learning algorithm to select the core genes in the NLR signal. TRIP6 was found to be a core gene with a relatively high gene weight, so the subsequent experiments of this study will focus on this gene.

### Cell culture

We purchased classic GBM cell lines U87, U251, and LN229 from ATCC and the normal brain tissue cell line NHA from QINGQI (SHANGHAI) BIOTECHNOLOGY DEVELOPMENT COMPANY. All cells were cultured according to the supplier’s recommendations in DMEM medium containing 10% FBS and 1% antibiotics, and were incubated in a 37°C incubator with 5% CO2.

### Quantitative reverse transcription polymerase chain reaction

We used the SevenFast Total RNA Extraction Kit for Cells to extract RNA and the SevenFast Two Step RT & qPCR Kit for reverse transcription and PCR. The procedures were performed according to the instructions provided in the reagent manuals. GAPDH and TRIP6 primers were provided by Sangon Biotech, with the specific primer sequences detailed in **Table 2**.

**Table 2:** Primer sequences.

	Forward primer	Reverse primer
TRIP6	ACGCCGAGATAGACTTGCTG	CTGGCTCTCTGGCTCCTA
CCL18	CTCTGCTGCCTCGTCTATACCT	CTTGGTTAGGAGGATGACACCT
AEBP1	GGGAGGACTATGAGGACTTTGAG	CGGAGGAGGCCCAAAGTAATAG
RARRES2	AAAGTCAGGCCCAATGGGAG	GGAAGTAGAAGCTGTGGGGG
SPP1	GCCGAGGTGATAGTGTGGTT	AACGGGGATGGCCTTGTATG
GAPDH	ACGGGAAGCTTGTCATCAAT	TGGACTCCACGACGTACTCA

### Immunohistochemistry

After dewaxing with xylene, the tissue sections were hydrated using different concentrations of alcohol. Following antigen retrieval and serum blocking, the sections were washed with PBS and then incubated overnight at 4°C with the primary antibody against TRIP6. On the following day, the sections were brought to room temperature, and secondary antibody and DAB chromogenic solution were applied. The staining was observed under a microscope. Hematoxylin counterstaining was performed, followed by washing with tap water, differentiation with hydrochloric acid alcohol, dehydration with increasing concentrations of alcohol, clearing with xylene, and finally mounting.

### Cell transfection

When the tumor cell density in the six-well plate reaches about 60%-80%, transfection is performed. The transfection reagent GP-transfect-Mate and siRNA are mixed in 200µL Opti-MEM. After standing for 5 minutes, mix them well. Then stand for another 15 minutes before adding them to the six-well plate. Of note, the culture medium should be replaced with complete medium after 5 hours, and the knockdown efficiency should be evaluated after 48 hours. The sequence of TRIP6 siRNA is shown in **Table 3**.

**Table 3:** siRNA interference sequences for TRIP6 gene knockdown.

	Sense (5’-3’)	Antisense (5’-3’)
GAPDH Positive control	UGACCUCAACUACAUGGUUTT	AACCAUGUAGUUGAGGUCATT
SiRNA-1:TRIP6-homo-1078	GCUGCUUUGUAUGUUCUACTT	GUAGAACAUACAAAGCAGCTT
SiRNA-2:TRIP6-homo-1337	GGACUUUCACAGGAAGUUUTT	AAACUUCCUGUGAAAGUCCTT

### Cell counting kit-8

After cell transfection, seed the tumor cells at a density of 3000 cells per well in a 96-well plate and incubate under 5% CO2 at 37°C. Perform CCK-8 assays (ab228554, Abcam) to measure the OD values at 450 nm at 24, 48, and 72 hours after plating.

### Wound healing assay

24 hours after transfection in a six-well plate, when the cell density is approximately 80%, perform the wound healing assay. Use a sterile 1 mL pipette tip to draw a straight line vertically across the cell monolayer. Wash the wells three times with PBS along the well wall to remove as many cell debris or floating cells as possible, and then add DMEM medium with 2% FBS. Incubate the cells in a 37°C incubator with 5% CO2. Observe and photograph the cell migration in the wound area at 0 hours, 24 hours, and 48 hours post-wounding using a microscope.

### RNA sequencing following TRIP6 gene knockdown

Lentivirus containing the SiRNA-1 (TRIP6-homo-1078) sequence was purchased from GenePharma and used to infect U251 cells, achieving TRIP6 gene knockdown. RNA sequencing was then performed on the knockdown cell line, with the sequencing process completed by Novogene. The general workflow included: strict quality control of RNA samples using the Agilent 2100 Bioanalyzer, enrichment of mRNA from total RNA using Oligo dT magnetic beads, synthesis of cDNA with random hexamer primers to construct the library, and final sequencing performed on the Illumina NovaSeq XPlus platform.

## Results

### Multi-omics characteristics of NLR signaling in GBM

**Figure 1:**
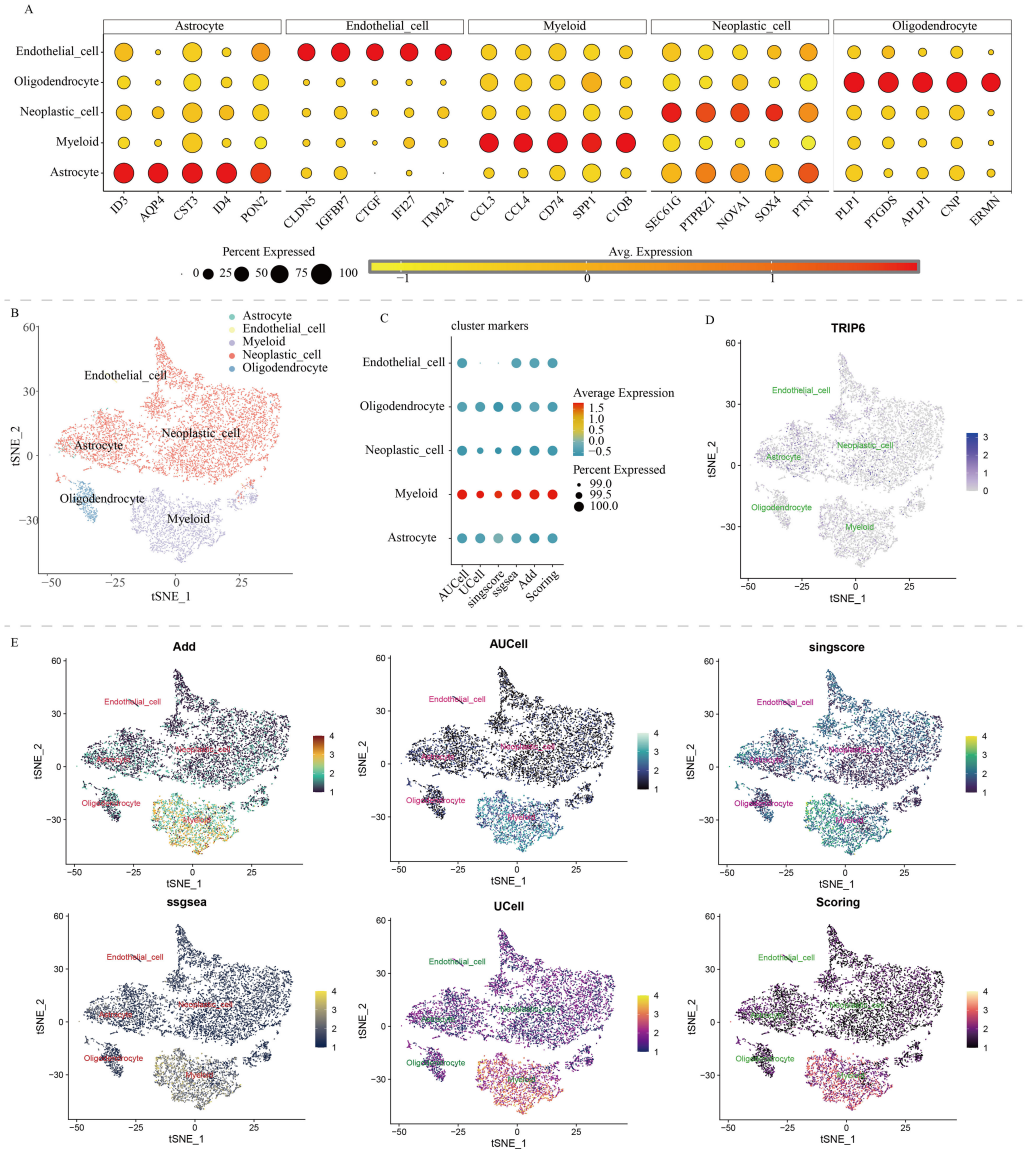
Single-cell analysis of NOD-like receptor signaling in GBM. **(A)** Cell annotation and cell marker. **(B)** t-SNE algorithm-based cell dimensionality reduction plot. **(C)** Quantitative analysis of the activity level of NOD-like receptor signaling in each cell type. **(D)** Single-cell expression profile of the TRIP6 gene. **(E)** Single-cell distribution of NOD-like receptor signaling activity in each cell type.

**Figure 2:**
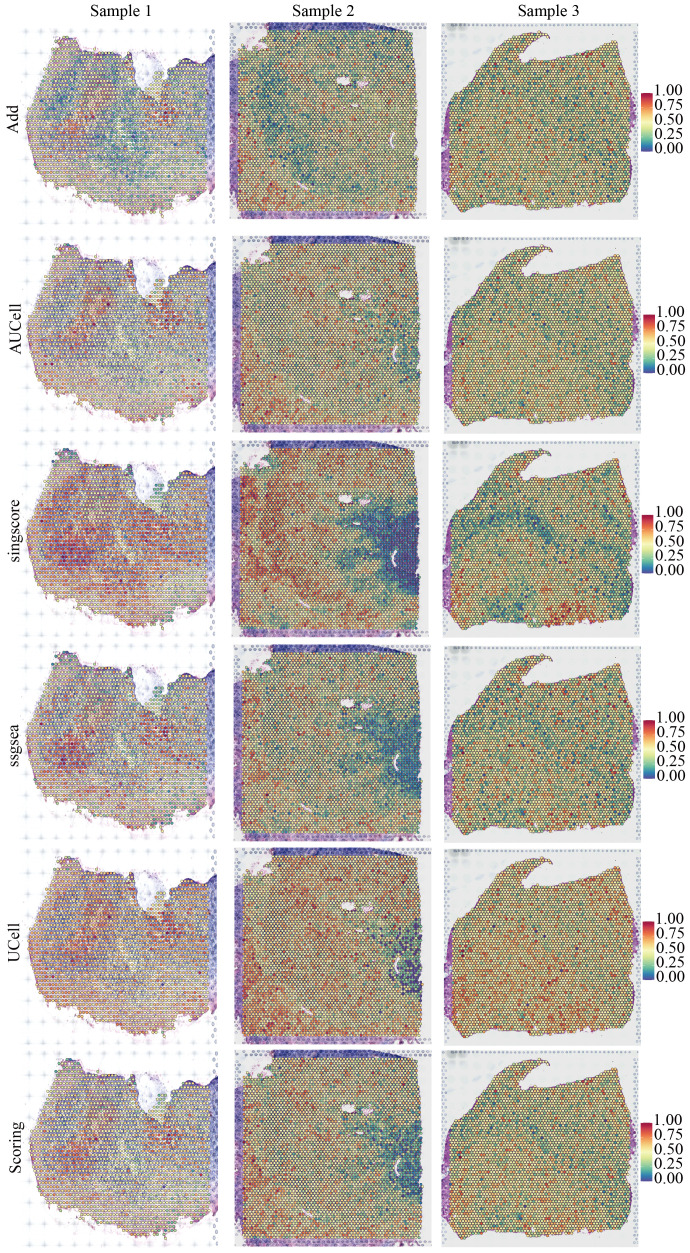
Spatial distribution characteristics of NOD-like receptor signaling in three pancreatic cancer samples.

We downloaded and processed single-cell sequencing data from 9 GBM samples, which included a total of 19,318 cells and 16,291 genes. After filtering out doublets using the DoubletFinder package, 416 doublets were removed. By controlling the number of UMIs per cell, the number of genes detected per cell, and mitochondrial content, we obtained 18,075 quality-controlled single cells for analysis (**Supplementary Figure S1**). Based on classical cell markers, these cells were classified into five types: Astrocyte, Endothelial cell, Myeloid, Neoplastic cell, and Oligodendrocyte (**Figure 1A**). The t-SNE dimensionality reduction clearly showed distinct boundaries between these cell subgroups, indicating accurate cell annotation and clustering results (**Figure 1B**). To reveal the characteristics of NLR signaling in different cell types, we applied six classic single-cell pathway enrichment analysis algorithms. The results consistently indicated that myeloid cells exhibited the most active NLR signaling (**Figures 1C, E**). Additionally, we analyzed spatial transcriptomics data in a similar manner. The results showed that different spatial locations in GBM patients might be associated with varying levels of NLR signal activation, suggesting a potential spatial distribution of myeloid cells (**Figure 2**).

### Identification of clinical heterogeneity in GBM patients based on NLR signaling

**Figure 3:**
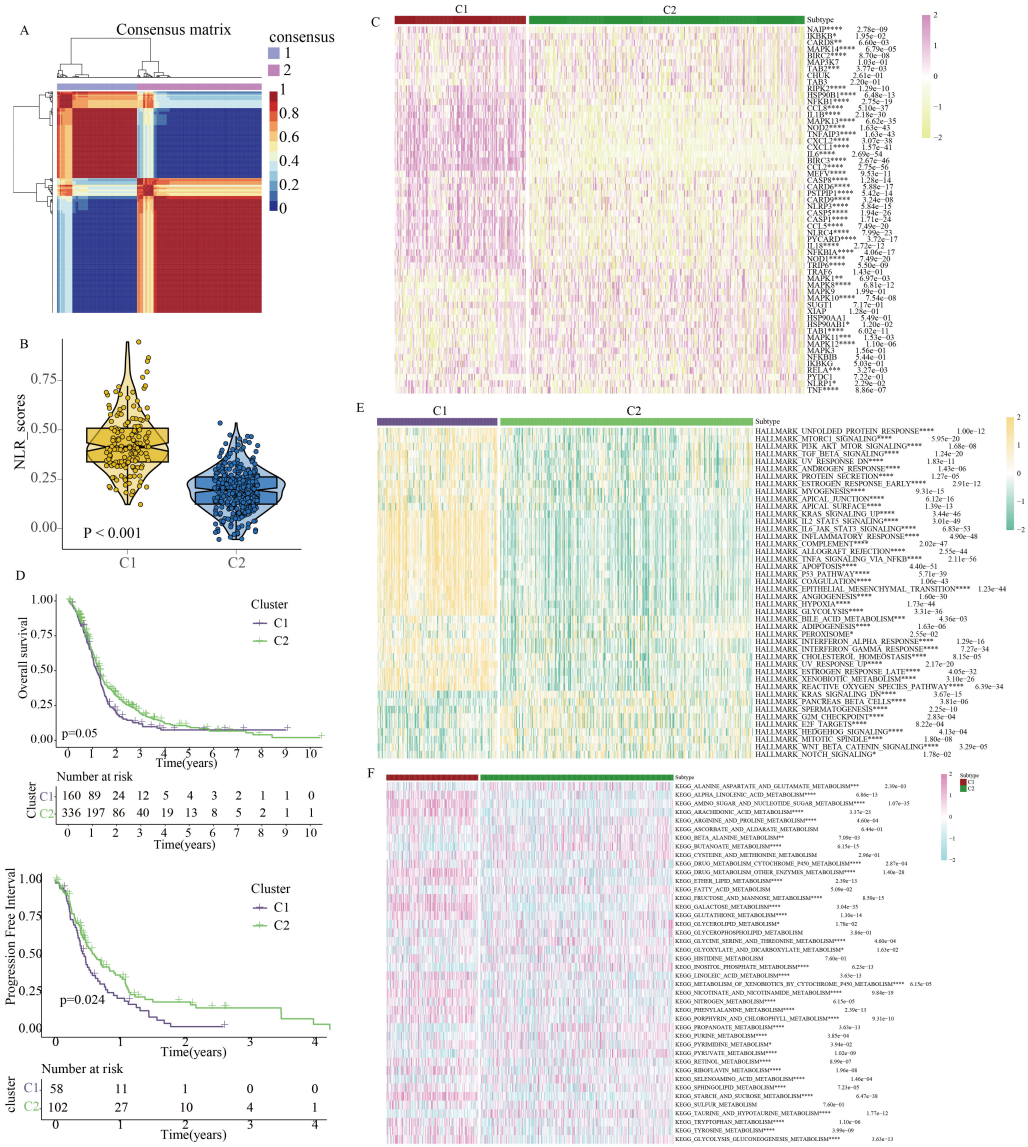
Identification of clinical heterogeneity and metabolic characteristics in GBM patients based on molecular features of NOD-like receptor signaling. **(A)** GBM molecular subtypes classified by the NMF algorithm. **(B)** Quantitative analysis of NOD-like receptor signaling activity between the two subtypes. **(C)** Expression profiles of NOD-like receptor signaling-related genes between the two subtypes. **(D)** Differences in prognostic indicators between the two subtypes. **(E)** Quantitative analysis of HALLMARK-related tumor signaling pathway activity between the two subtypes. **(F)** Quantitative analysis of KEGG-related metabolic signaling pathway activity between the two subtypes. (*p < 0.05, **p < 0.01, ***p < 0.005, ****p < 0.001).

Based on the expression profile of NLR signaling genes, we successfully utilized the NMF clustering algorithm to classify GBM patients into two clinical subtypes: C1 and C2 (**Figure 3A**). Single-sample gene set enrichment analysis revealed that C1 GBM patients had higher NLR signaling scores compared to C2 patients (**Figure 3B**). We then compared the expression of NLR-related genes between the two GBM subtypes, finding that most NLR genes, including TRIP6, NOD1, NLRP3, and NOD2, were more highly expressed in the C1 subtype (**Figure 3C**). Kaplan-Meier survival analysis indicated that C1 GBM patients had poorer clinical outcomes compared to C2 patients (**Figure 3D**). To investigate the potential reasons for the prognostic differences between the two GBM subtypes, we analyzed tumor-related classic signaling pathways and the immune microenvironment. Gene set enrichment analysis based on HALLMARK signaling pathways revealed that many pathways associated with cancer development and progression, such as PI3K-AKT signaling, TGF-β signaling, Kras signaling, angiogenesis signaling, and glycolysis signaling, were aberrantly active in C1 GBM patients (**Figure 3E**). More importantly, C1 and C2 patients showed obvious difference in activities of metabolism pathways (**Figure 3F**). In summary, the activation of these aberrant signaling pathways might be a potential factor contributing to the different prognoses observed between C1 and C2 GBM patients.

**Figure 4:**
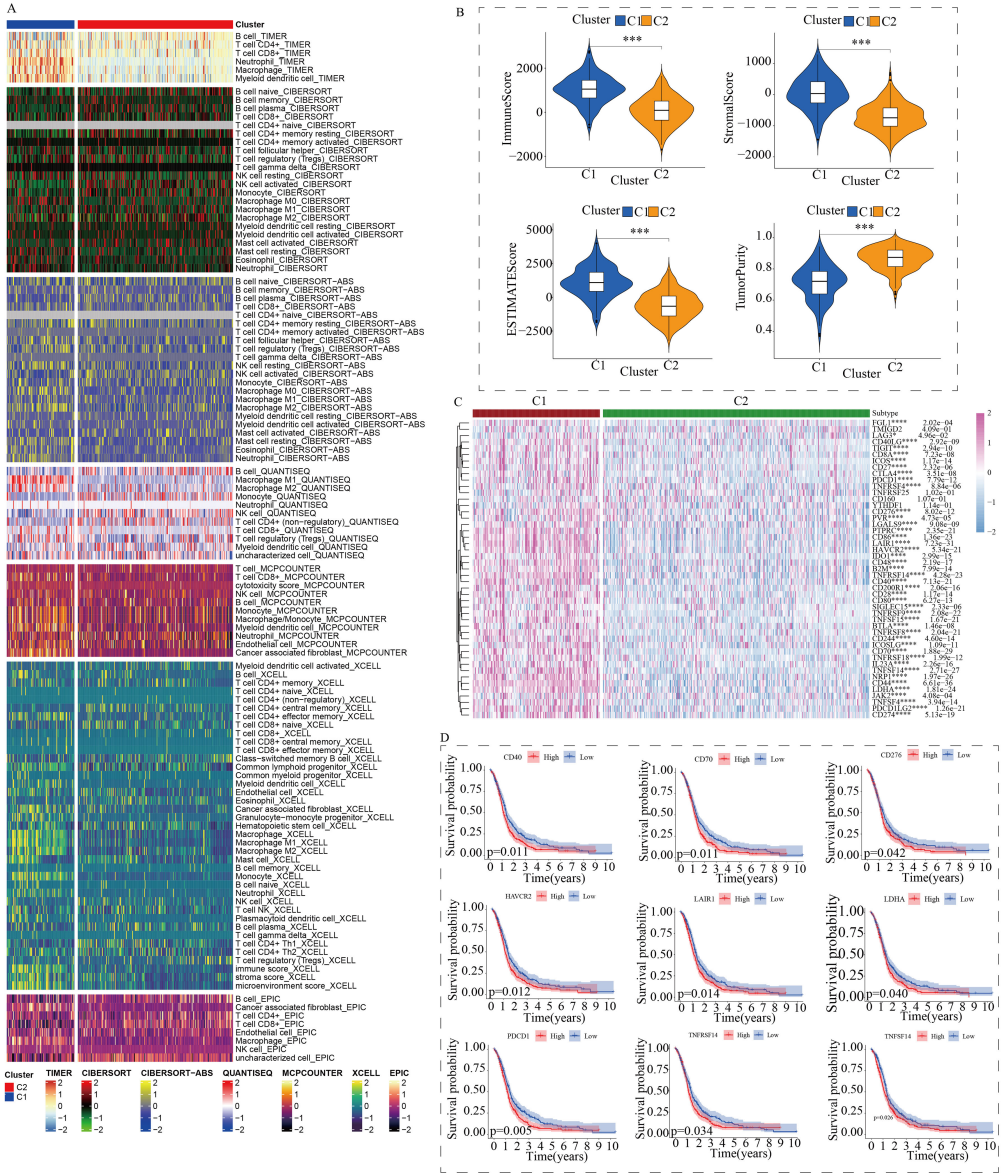
Differences in the immune microenvironment between the two subtypes. **(A)** Assessment of immune cell infiltration between the two subtypes using a pan-immune genomic algorithm. **(B)** Evaluation of immune status differences between the two subtypes using the ESTIMATE algorithm. **(C)** Quantitative analysis of the expression differences of immune checkpoint genes between the two subtypes. **(D)** Prognostic impact of immune checkpoints in GBM patients. (*p < 0.05, ***p < 0.005, ****p < 0.001).

**Figure 5:**
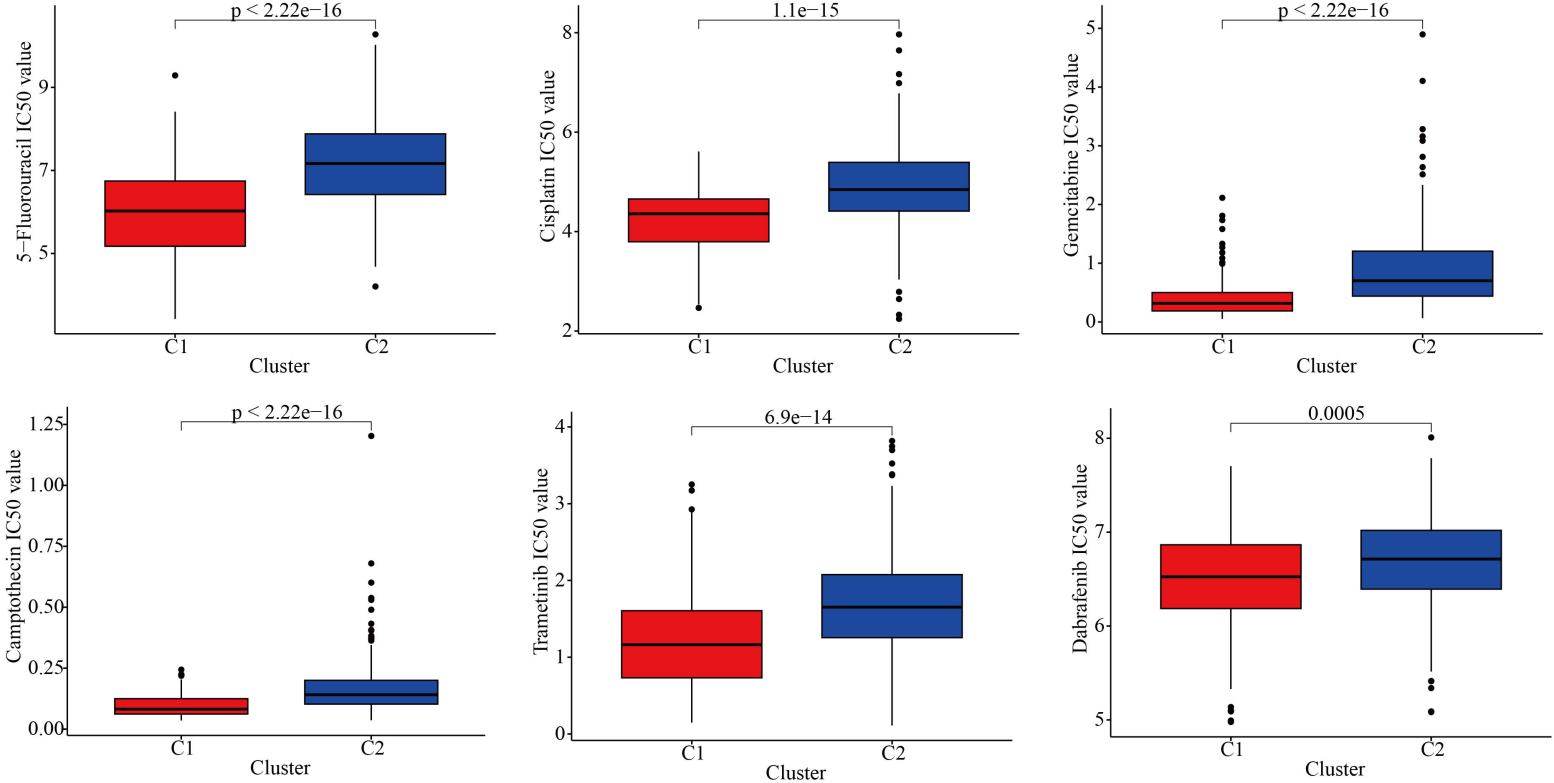
Drug sensitivity differences between the two subtypes.

To further explore the discrepancies in the immune microenvironment, we used various immunocyte-predicted algorithms, including TIMER, CIBERSORT, QUANTISEQ, XCELL, and EPIC. As shown in **Figures 4A** and **B**, the results indicated that C1 subtype exhibited higher levels of immune cell infiltration compared to C2 subtype. The immune microenvironment is a comprehensive and complex topic. Simply analyzing the level of immune cell infiltration is far from enough to explain the problem. Therefore, we also analyzed the levels of immune checkpoints. Immune checkpoints are the determining factors for immune cells to exert their anti-tumor functions. The results showed that almost all immune checkpoint genes were abnormally overexpressed in patients with C1 subtype (**Figure 4C**). Abnormally high expression of immune checkpoints often indicated poor prognosis in GBM patients (**Figure 4D**). In addition, we also found that some targeted drugs might be effective for patients with C1 subtype with poor prognosis (**Figure 5**).

**Figure 6:**
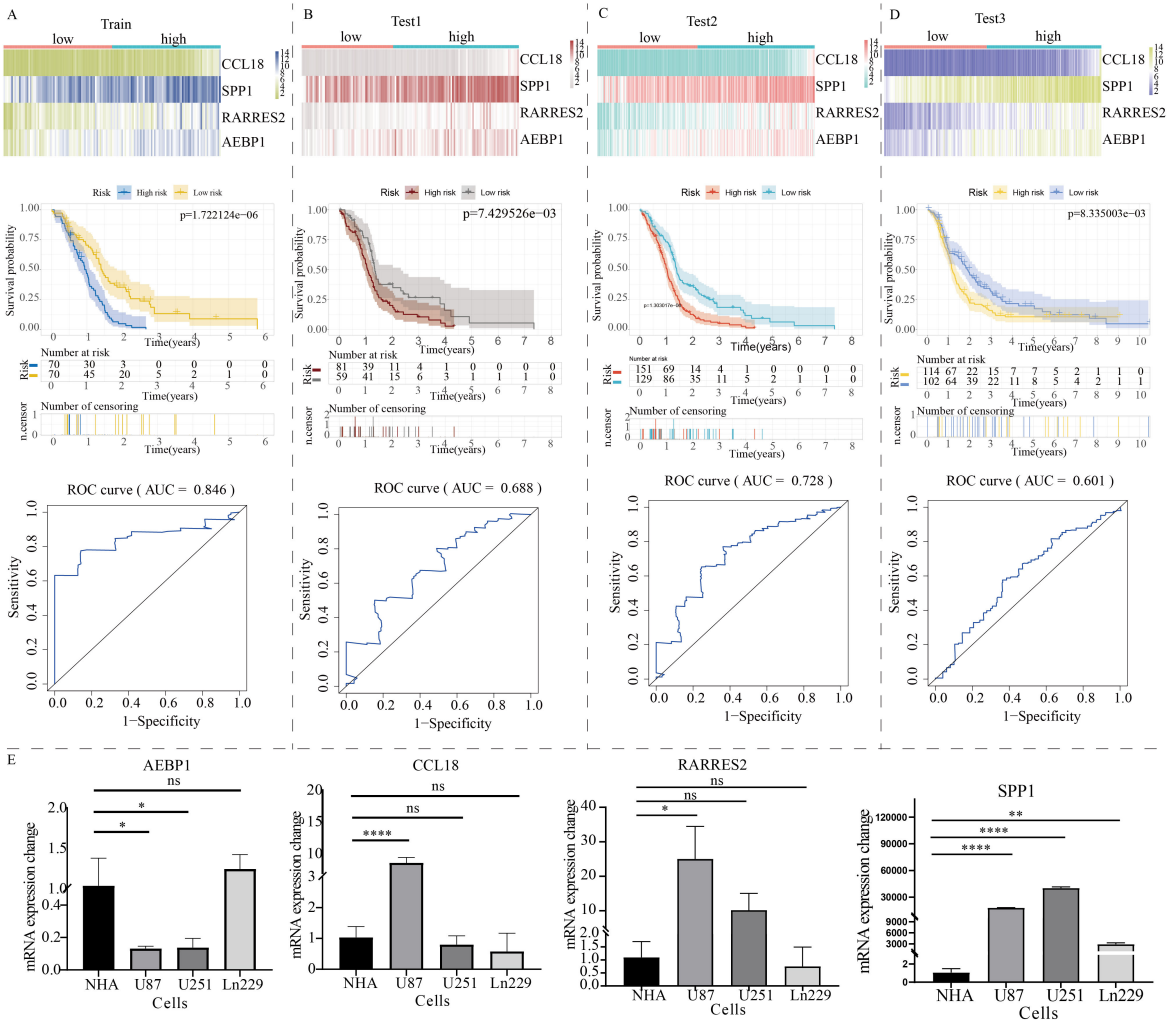
Construction of a prognostic model for GBM patients based on the expression profile of NOD-like receptor signaling. The prognostic performance of the model in **(A)** the train group, **(B)** test1 group, **(C)** test2 group, and **(D)** test3 group. The top section shows the expression distribution characteristics of model genes, the middle section presents the KM curve revealing the prognostic differentiation of the model, and the bottom section displays the ROC curve revealing the prognostic accuracy of the model. **(E)** The PCR results of model gene expression. (*p < 0.05, **p < 0.01, ****p < 0.001). ns, no significance.

The volcano plot revealed differentially expressed genes between C1 and C2 GBM subtypes (**Supplementary Figure S2A**). KEGG enrichment analysis showed that these differentially expressed genes were mainly enriched in cytokine signaling, cell adhesion, extracellular matrix remodeling, and TNF signaling (**Supplementary Figure S2B**). Subsequently, these differentially expressed genes were used to construct a novel NLR signaling-related prognostic model. LASSO regression analysis identified 8 potential candidate molecules (**Supplementary Figure S3A**), and multi-factor Cox regression analysis was performed to develop the model, resulting in a GBM prognostic marker composed of 4 genes (**Supplementary Figure S3B**). These four genes are: CCL18, SPP1, RARRES2, and AEBP1. The novel prognostic marker demonstrated substantial and stable prognostic performance across the training set, internal validation set, and external validation set (**Figures 6A–D**). The expression patterns of these four model genes (i.e. CCL18, SPP1, RARRES2, and AEBP1) in glioma cells were shown in **Figure 6E**. Similarly, low and high subgroups showed obvious difference in activities of metabolism pathways (**Supplementary Figure S4**).

### TRIP6 gene regulates the malignant biological behavior of GBM

**Figure 7:**
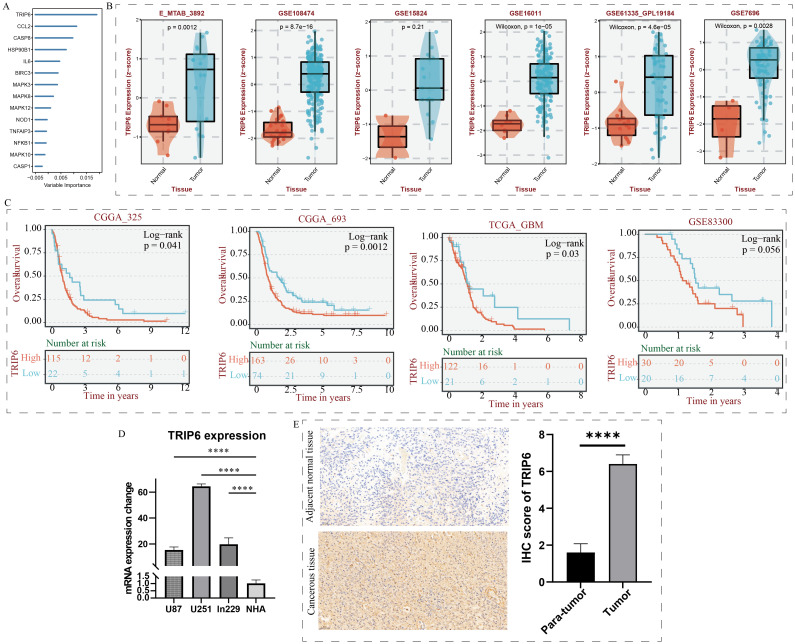
Identification and expression validation of the core gene TRIP6. **(A)** Calculation of NOD-like receptor signaling-related importance using the random forest algorithm. **(B)** Association between TRIP6 expression and clinical characteristics. **(C)** Association between TRIP6 expression and prognosis in GBM patients. **(D)** qRT-PCR experiment validating the abnormal expression of the TRIP6 gene at the cellular level. **(E)** Immunohistochemistry experiment validating the expression profile of the TRIP6 gene. (****p < 0.001).

**Figure 8:**
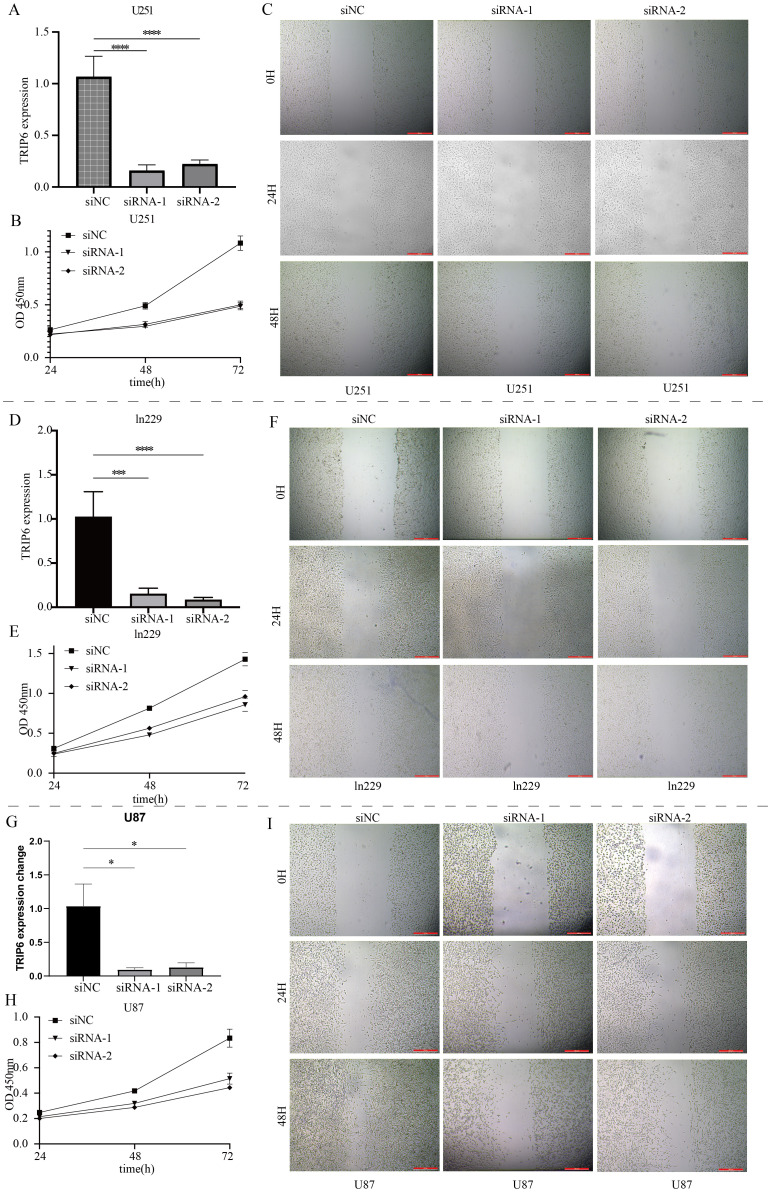
Functional validation of the core gene TRIP6. **(A)** Knockdown results of TRIP6 in U251 cells. **(B)** Effect of TRIP6 knockdown on U251 cell proliferation (CCK8 assay). **(C)** Effect of TRIP6 knockdown on U251 cell migration (wound healing assay). **(D)** Knockdown results of TRIP6 in LN229 cells. **(E)** Effect of TRIP6 knockdown on LN229 cell proliferation (CCK8 assay). **(F)** Effect of TRIP6 knockdown on LN229 cell migration (wound healing assay). **(G)** Knockdown results of TRIP6 in U87 cells. **(H)** Effect of TRIP6 knockdown on U87 cell proliferation (CCK8 assay). **(I)** Effect of TRIP6 knockdown on U87 cell migration (wound healing assay). (*p < 0.05, ***p < 0.005, ****p < 0.001).

As shown in **Figure 7A**, random forest analysis revealed that the TRIP6 gene ranked as the most significant among all NLR signaling genes. Therefore, this study focused on the expression traits and functional roles of TRIP6 in GBM. Firstly, we observed that TRIP6 gene expression was significantly higher in GBM tissues compared to normal tissues (**Figure 7B**) and was closely associated with poor prognosis in GBM patients. We also mapped the single-cell signature of the TRIP6 gene (**Figure 1D**). Abnormally high expression of TRIP6 gene was closely associated with poor prognosis in GBM patients (**Figure 7C**). PCR results confirmed that TRIP6 expression was markedly elevated in U87, U251, and Ln229 cell lines compared to NHA cells (**Figure 7D**). Immunohistochemistry results also showed that TRIP6 protein expression in GBM tissue was significantly higher than that in adjacent normal brain tissue (**Figure 7E**). We then conducted TRIP6 knockdown experiments, which successfully reduced TRIP6 expression in all three glioma cell lines (**Figures 8A, D, G**). Knockdown of TRIP6 inhibited cell proliferation of U87, U251, and Ln229 cell lines (**Figures 8B, E, H**). Additionally, scratch assay results demonstrated that knockdown of TRIP6 suppressed cell migration of U87, U251, and Ln229 cell lines (**Figures 8C, F, I**).

**Figure 9:**
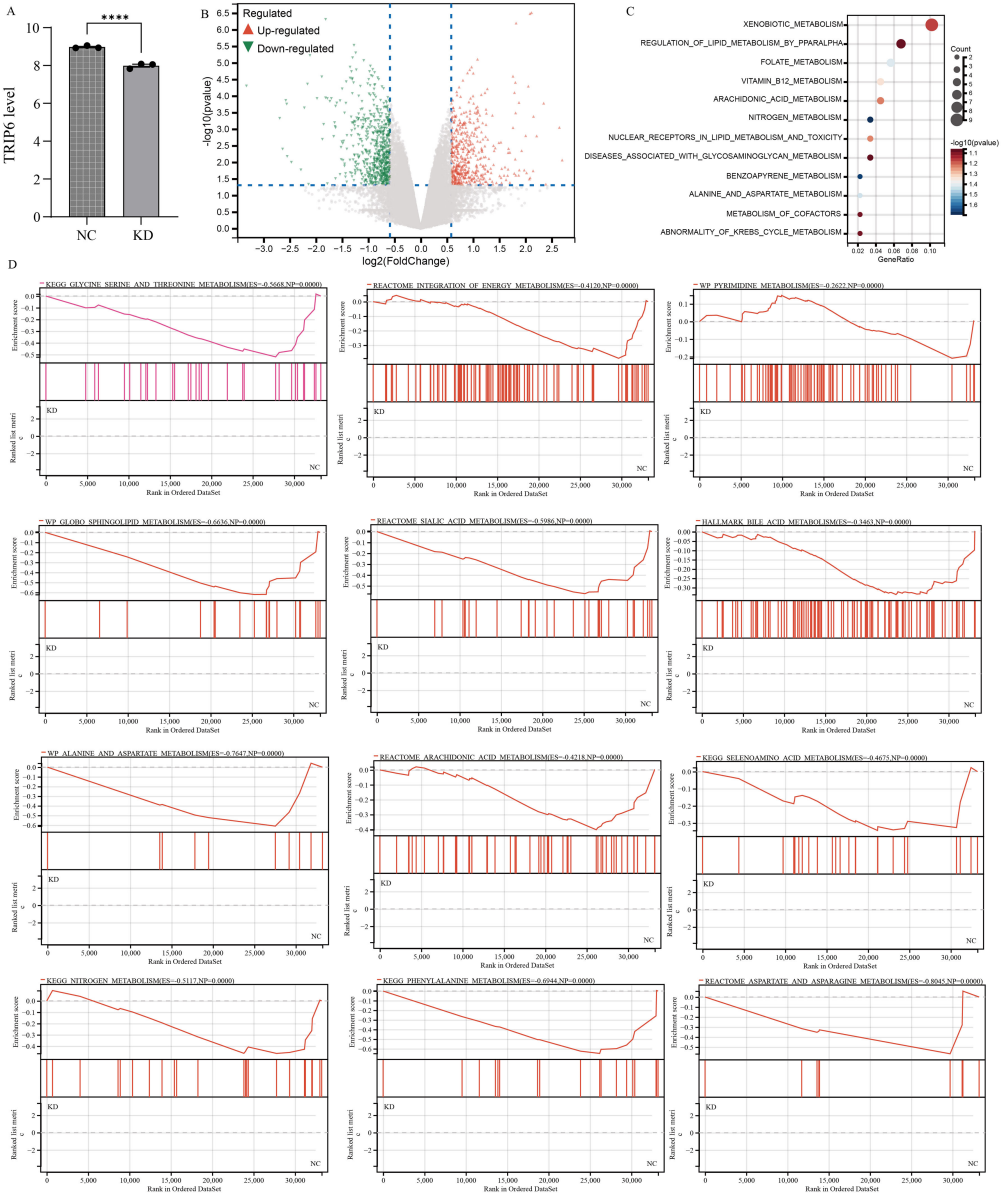
RNA sequencing following TRIP6 gene knockdown. **(A)** Validation of TRIP6 expression; **(B)** Volcano plot illustrating the differentially expressed genes after TRIP6 knockdown in U251 cells; **(C)** Pathway enrichment analysis of differentially expressed genes; **(D)** GSEA analysis of the impact of TRIP6 on metabolic pathways. (****p < 0.001).

To investigate the impact of TRIP6 gene on downstream pathways, we obtained stable knockdown of TRIP6 in U251 cells using lentiviral infection. The RNA sequencing data has been uploaded as **Supplementary Table S1**. Subsequent analysis of sequencing data further confirmed the successful knockdown of TRIP6 gene (**Figure 9A**). Volcano plot revealed that knockdown of TRIP6 gene significantly affected the expression levels of downstream genes (**Figure 9B**). Besides, enrichment analysis revealed that downstream genes were predominantly associated with metabolic pathways (**Figure 9C**), suggesting that TRIP6 played a critical regulatory role in GBM metabolic reprogramming. GSEA analysis demonstrated that TRIP6 knockdown led to the suppression of numerous lipid metabolism and amino acid metabolism pathways (**Figure 9D**). This process affected energy metabolism in GBM microenvironment. In summary, TRIP6 might promote GBM progression by regulating energy metabolism pathways.

## Discussion

GBM is a highly aggressive primary brain tumor with a poor prognosis. Its significant heterogeneity and therapeutic resistance limit the efficacy of targeted therapies, highlighting the urgent need for novel molecular biomarkers. These biomarkers can provide insight into the biology of GBM, uncover potential therapeutic targets and contribute to the development of new treatment strategies. In this study, we explored the biological role of TRIP6, a key gene in the NLR signaling pathway, in GBM. Using single-cell sequencing data from GBM, we observed that NLR signaling was primarily active in myeloid cells. Spatial transcriptome analysis further revealed that the expression of NLR signaling pathway genes was different in different histological regions of GBM tumors. Transcriptome cluster analysis also showed that groups with different levels of NLR signaling gene expression showed different sensitivities to classical chemotherapy drugs. Through bioinformatics analysis of mRNA sequencing data, we identified TRIP6 as a key gene in the NLR signaling pathway. TRIP6 expression was abnormally elevated in GBM tumor tissues and is associated with poor prognosis. This finding was confirmed by *in vitro* experiments and clinical sample analysis. To investigate the role of TRIP6 in GBM progression, we conducted TRIP6 gene silencing experiments. The results showed that silencing TRIP6 expression could significantly inhibit the proliferation and migration of tumor cells.

Recent research has revealed that NLRs played a crucial role in innate immunity and inflammatory responses (26, 27). They might influence the formation and evolution of the tumor microenvironment by regulating inflammatory responses. It is worth noting that the research on NLR family including NLRP3 inflammasome in tumor targeted therapy has made significant progress (28–30). Recent studies (31–35) on the tumor microenvironment of glioma have highlighted that myeloid cells, including bone marrow-derived macrophages, microglia, myeloid-derived suppressor cells, dendritic cells, and neutrophils, play important roles. These cells can regulate the tumor microenvironment, suppress anti-tumor immune responses, and directly promote tumor cell growth and invasion. These findings made myeloid cells a breakthrough direction for glioma treatment. Emerging evidence indicated that gliomas might promote the growth of tumor cells and their spread along cerebral blood vessels by destroying the blood-brain barrier and secreting a variety of pro-angiogenic factors (36, 37). Shivanjali et al. (38) discovered that NLR signaling pathway affected expression and function of various downstream molecules (e.g. IL-1β, IL-18, and caspase-1). These molecules induced the expression of angiogenic factors (e.g. VEGF, FGF, and HIF-1α), thereby promoting angiogenesis. After integrating and analyzing transcriptome data and clinical information of GBM patients, TRIP6 was considered as the hub gene within NLR signaling pathway. TRIP6 had the potential to influence the proliferation and migration of glioma cell, thereby causing unfavorable clinical outcomes.

Previous studies have identified TRIP6 as a cytoskeletal protein involved in various cellular processes, including cell motility, anti-apoptotic signaling, and transcriptional regulation (39–41). TRIP6 functioned as an oncogenic role in various solid tumors via regulation of multiple tumor-associated signaling pathways, such as Wnt/β-catenin (42–46). Our study also found that high expression of TRIP6 was associated with significant immune cell infiltration. Similar phenomenon has reported in the Liu et al. study (47) that expression level of TRIP6 was closely associated with glycolysis and immunocyte infiltration. TRIP6 might serve as a potential biomarker for prediction of immune microenvironment in GBM patients, and provide insights for immunotherapy strategies. Recent studies have shown that TRIP6 was significantly upregulated in paclitaxel-resistant tumor cells (48, 49), but the specific mechanism remained unclear. Additionally, aberrant expression of TRIP6 gene was associated with metastasis and drug resistance of colorectal cancer (42).

In order to detect the downstream signals of TRIP6 in GBM, RNA sequencing was carried out after silencing TRIP6 expression in glioma cell. A series of metabolism-related pathways were obviously inhibited following TRIP6 silencing. These metabolism pathways were primarily associated with tumor energy metabolism. Of note, our study firstly reported the association of TRIP with energy metabolism.

There are some limitations that need to be noted. We did not study the association between TRIP6 gene expression and glioma drug resistance. In addition, we have not conducted in-depth experimental verification on the specific regulatory mechanism of TRIP6 gene on glioma energy metabolism. The NLR signal prognostic markers developed in this study need to be verified by more clinical cohorts before they can be truly put into clinical practice.

In summary, we used single-cell methods to characterize the cell subsets within GBM and performed spatial analysis of NLR signaling pathway expression in tissue slices. Through transcriptome sequencing data, we identified TRIP6 as a key gene in the NLRs pathway involved in GBM development. *In vitro* knockdown experiments showed that TRIP6 promoted the proliferation and metastasis of GBM, and its high expression was associated with poor prognosis. TRIP6 can be used as a novel biomarker for predicting GBM prognosis, tumor metastasis, and chemotherapy response, assisting in monitoring tumor progression and adjusting treatment strategies.

## Data Availability

The original contributions presented in the study are included in the article/**Supplementary Material**. Further inquiries can be directed to the corresponding authors.

## Supplementary material

Supplementary Table 1 RNA sequencing data following TRIP6 gene knockdown.

Supplementary Figure 1 Quality control plots of single-cell data. **(A)** Correlations between nCount_RNA, nFeature_RNA, mitochondrial content, and ribosomal content. **(B)** Distribution range of Count_RNA, nFeature_RNA, mitochondrial content, and ribosomal content for each glioma sample.

Supplementary Figure 2 Differential molecules and pathway enrichment between the two subtypes. **(A)** Differentially expressed genes between subtypes C1 and C2 (volcano plot format). **(B)** Functional enrichment analysis of differentially expressed genes.

Supplementary Figure 3 LASSO-Cox regression analysis. **(A)** LASSO regression analysis to filter candidate variables. **(B)** Multivariate Cox regression analysis to construct a prognostic model.

Supplementary Figure 4 Differences in metabolic characteristics between high- and low-risk groups of GBM patients in the four cohorts. (*p < 0.05, **p < 0.01, ***p < 0.005, ****p < 0.001).
