# Supplementary figures and images for: Characterization of NOD-like receptor-based molecular heterogeneity in glioma and its association with immune micro-environment and metabolism reprogramming
Source: Front Immunol. 2025 Jan 15;15:1498583. doi: 10.3389/fimmu.2024.1498583 (PMC11774718; doi:10.3389/fimmu.2024.1498583)

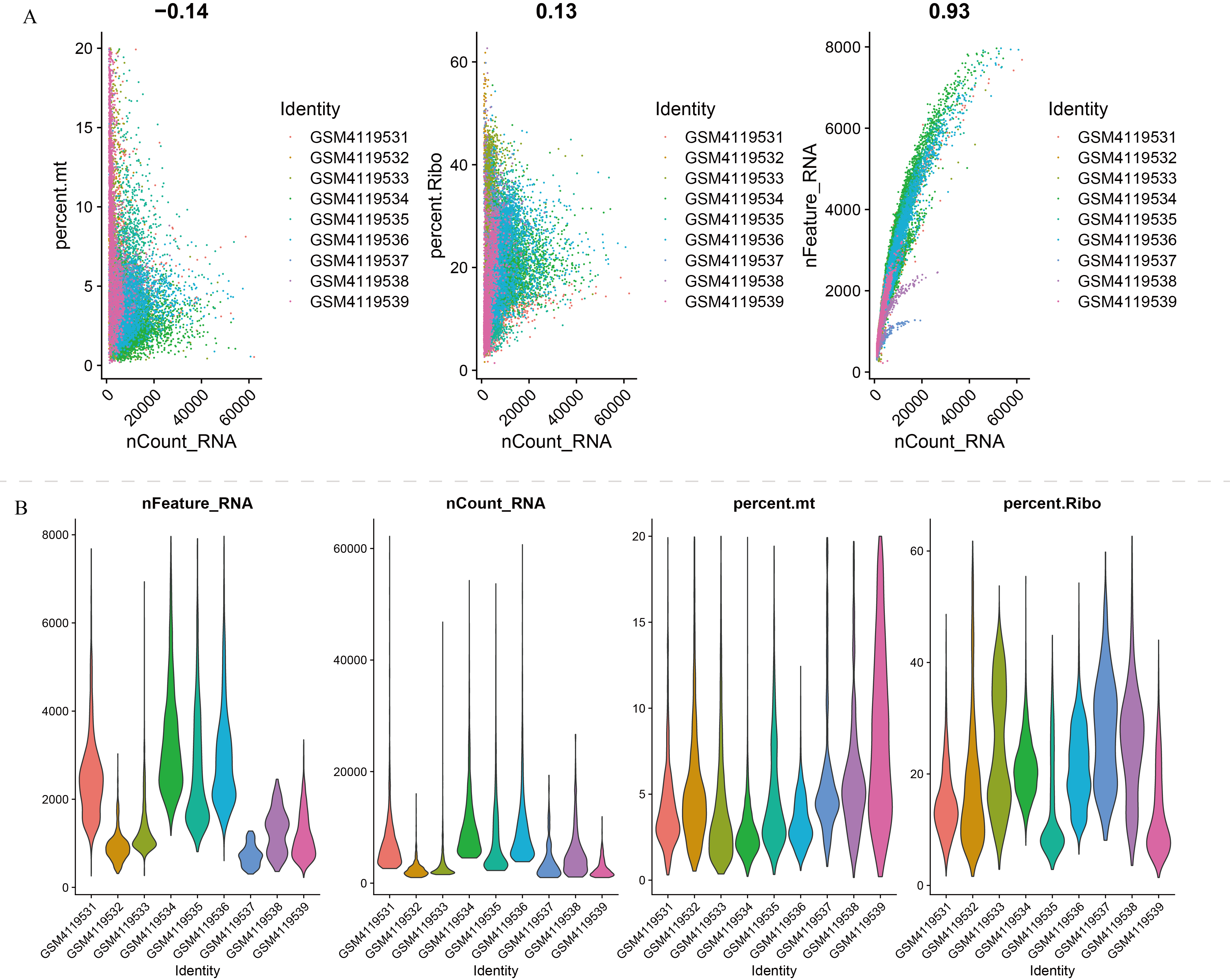

Supplement: Supplementary Figure 1 — Quality control plots of single-cell data. (A) Correlations between nCount_RNA, nFeature_RNA, mitochondrial content, and ribosomal content. (B) Distribution range of Count_RNA, nFeature_RNA, mitochondrial content, and ribosomal content for each glioma sample. [file Image1.jpeg]

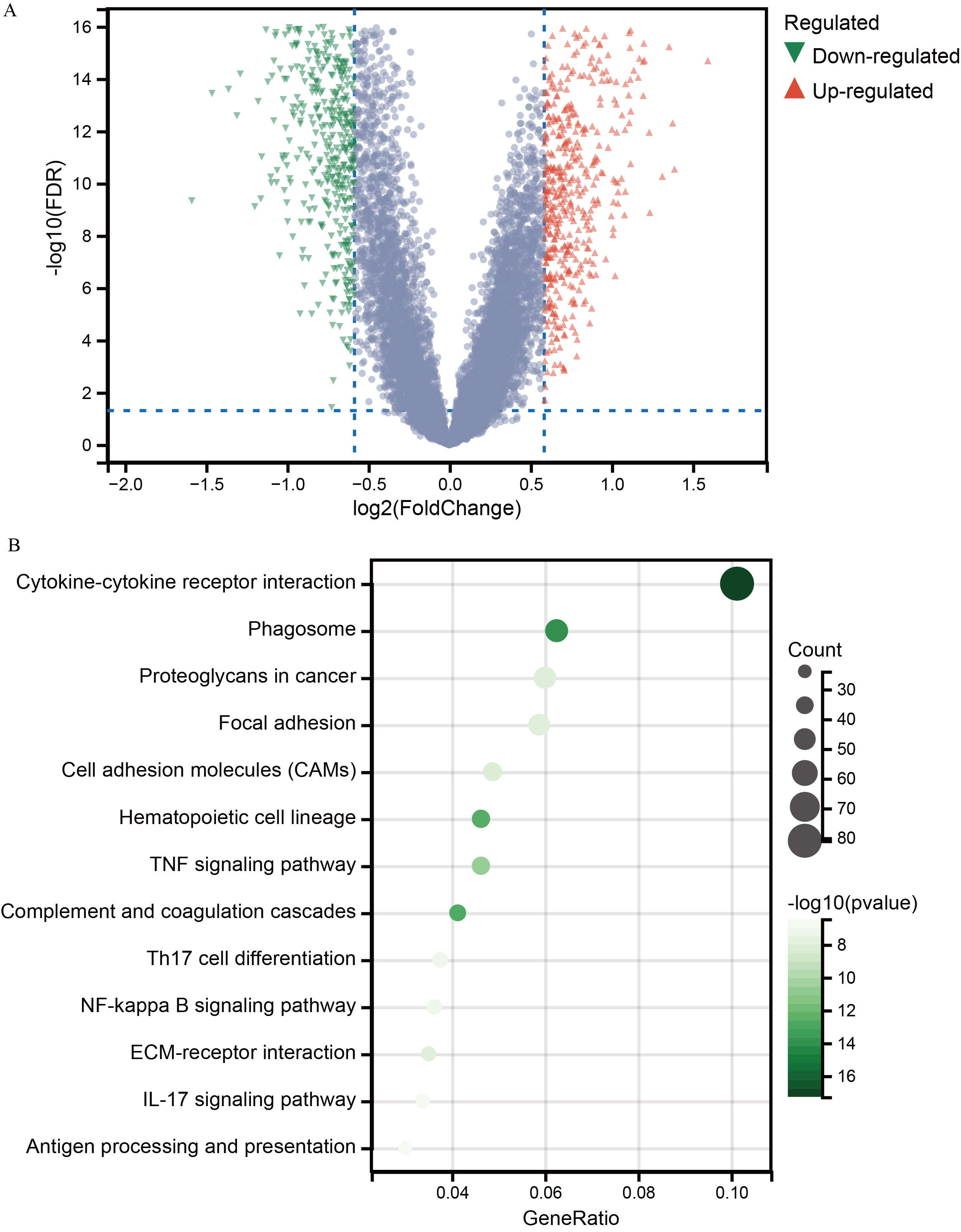

Supplement: Supplementary Figure 2 — Differential molecules and pathway enrichment between the two subtypes. (A) Differentially expressed genes between subtypes C1 and C2 (volcano plot format). (B) Functional enrichment analysis of differentially expressed genes. [file Image2.jpeg]

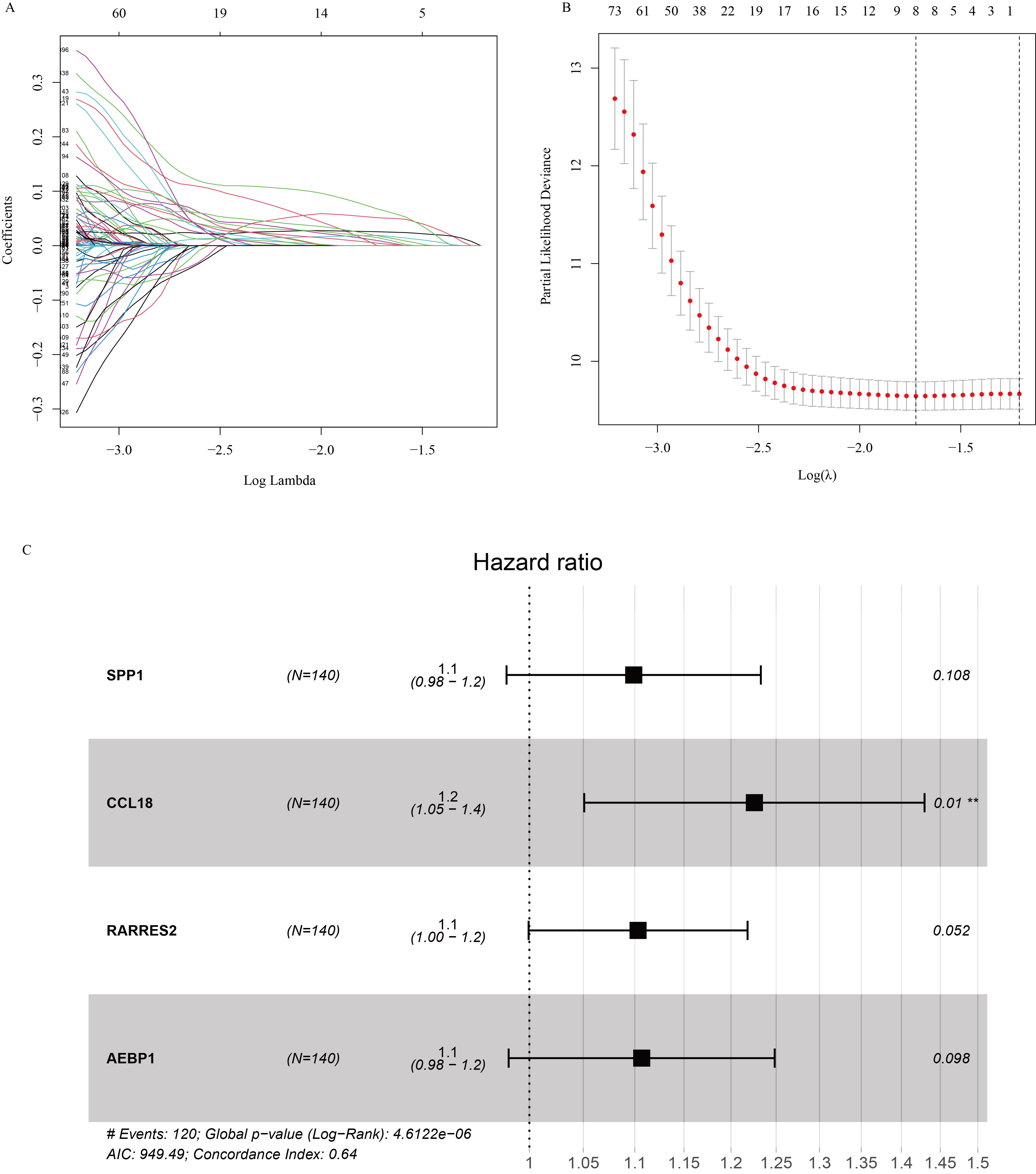

Supplement: Supplementary Figure 3 — LASSO-Cox regression analysis. (A) LASSO regression analysis to filter candidate variables. (B) Multivariate Cox regression analysis to construct a prognostic model. [file Image3.jpeg]

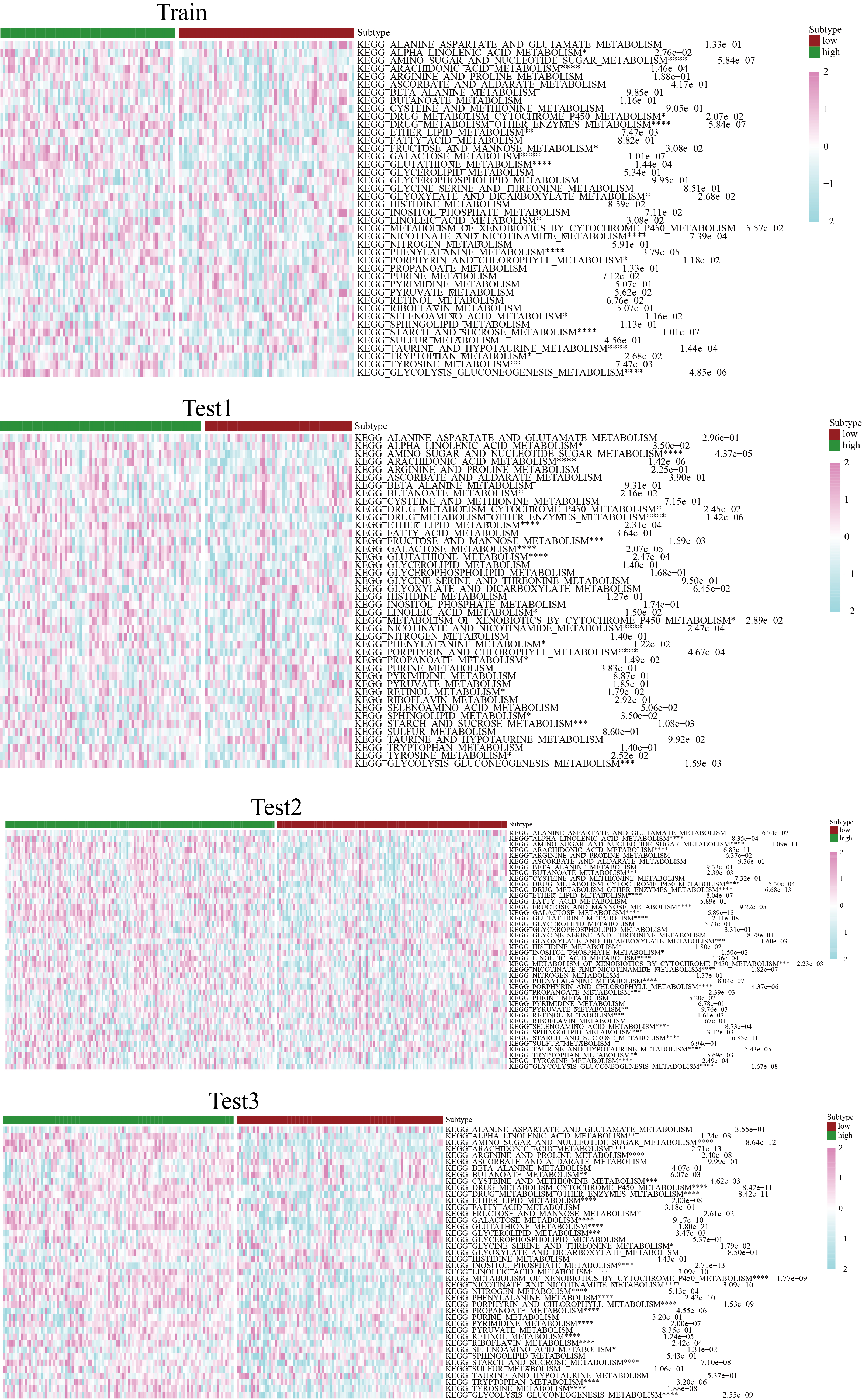

Supplement: Supplementary Figure 4 — Differences in metabolic characteristics between high- and low-risk groups of GBM patients in the four cohorts. (*p < 0.05, **p < 0.01, ***p < 0.005, ****p < 0.001). [file Image4.jpeg]
